# CRAC-DM: class relation-aware categorical diffusion model for surgical scene segmentation

**DOI:** 10.1007/s11548-026-03601-7

**Published:** 2026-04-13

**Authors:** Yihang Zhou, Chi Xu, Zaid Awad, Stamatia Giannarou

**Affiliations:** 1 Department of Surgery and Cancer, Hamlyn Centre for Robotic Surgery, Imperial College London, London, UK; 2 Imperial College Healthcare NHS Trust, London, UK

**Keywords:** Diffusion model, Surgical scene segmentation, Categorical noise, Class relation modeling

## Abstract

**Purpose:**

Accurate multi-class segmentation of surgical scenes remains challenging due to ambiguous anatomical boundaries and imaging artifacts. While diffusion-based segmentation methods have achieved good results, they rely on computationally heavy continuous diffusion processes. Recent discrete diffusion variants reduce computation but their performance is limited due to uniform noise, ignoring inter-class relationships that are crucial for generating semantically relevant training signals. To address this gap, we propose the class relation-aware categorical diffusion model (CRAC-DM).

**Methods:**

CRAC-DM consists of three key components. In the forward process, we embed semantic class relationships for the first time when adding categorical noise via a class relation-aware transition matrix, biasing noise toward semantically similar categories to generate class-aware supervision signals. In the reverse process, we introduce a step-skipping categorical denoiser (S2D) tailored for discrete diffusion segmentation, enabling fast inference. To further boost inference, we propose a novel confidence-adaptive test time augmentation (TTA) that selectively refines regions of interest with low prediction confidence using entropy-weighted aggregation.

**Results:**

The proposed CRAC-DM was evaluated on the publicly available CholecSeg8k and EndoVis18 datasets. It consistently outperformed state-of-the-art U-Net-, transformer-, and diffusion-based baselines, particularly on tissue segmentation, even for small and under-represented classes while significantly reducing inference time compared to diffusion baselines.

**Conclusion:**

By enhancing the forward process with inter-class similarity and improving the reverse process with a deterministic S2D and targeted TTA, CRAC-DM achieves superior segmentation accuracy, efficiency, and reliability, paving the way for practical deployment in computer-assisted surgery.

## Introduction

Accurate multi-class segmentation in surgical scenes is critical for computer-assisted surgery, as it delineates different tissue types and anatomical structures to support decision-making and improve patient care [1]. However, challenges such as ambiguous tissue boundaries, noise and occlusions can significantly degrade the performance of existing segmentation models [2].

Recently, diffusion-based approaches have emerged as a new paradigm for segmentation, building on their success in computer vision tasks such as image generation [3]. When applied to segmentation, diffusion models progressively perform denoising, effectively addressing issues such as noise and blurry boundaries [4]. This iterative process produces highly detailed segmentation maps, particularly beneficial for complex surgical scenes [5].

Additionally, the generative nature of diffusion models allows multiple plausible segmentation hypotheses through repeated sampling, making them well suited for medical scenarios characterized by inherent boundary uncertainty [4]. Unlike earlier uncertainty segmentation methods such as the probabilistic U-Net [6], which compress images into a low-dimensional latent vector that often limits output diversity, diffusion models avoid this information bottleneck by operating directly in the high-dimensional image space, improving segmentation accuracy in complex scenes and diversity. The above advantages explain the substantial segmentation gains achieved by diffusion models, even when using a simple backbone such as a U-Net [5, 7].

However, most diffusion-based segmentation methods rely on continuous Gaussian perturbations [4, 5], originally designed for natural image synthesis. Treating segmentation maps as continuous data introduces unnecessary computational overhead and potential numerical instability, while also randomly assigning distances between arbitrary class indices. A promising alternative is categorical diffusion, which operates directly in a discrete label space. For instance, BerDiff [8] employs Bernoulli noise for binary segmentation, while CCDM [7] applies probabilistic transitions between classes, facilitating categorical noise injection for multi-class scenarios. However, CCDM uses uniform noise, treating all class transitions as equally possible. This ignores the inherent class relationships where specific classes are far more related than others. For example, visually similar tissues and frequently interacting tools like the grasper and liver, have high semantic correlation. The model with uniform noise wastes capacity on learning transitions with low probability.

To address the above limitations, we propose the CRAC-DM framework for surgical scene segmentation. Our contributions are:

1. **Forward diffusion process.** We construct, for the first time, a dataset-level class relationship matrix that quantifies semantic similarity between classes, computed using pretrained image features. This matrix bias the categorical diffusion process so that noise transitions occur more frequently between semantically related classes, producing more realistic noisy images and class-aware supervision signals.
2. **Reverse denoising process.** We introduce the S2D sampler tailored for discrete diffusion segmentation that enables fast inference, and propose a novel confidence-adaptive TTA that selectively refines ROI with low class prediction confidence. Specifically, the model identifies these ROI based on the prediction entropy, applies geometric augmentations (e.g., flips and rotations) for them and fuses the results through entropy-weighted averaging. This design improves segmentation accuracy and reliability while reducing inference time.

We compare CRAC-DM against powerful U-Net-, transformer-, and diffusion-based baselines. Performance evaluation on the publicly available CholecSeg8k [9] and EndoVis18 [10] datasets demonstrates that CRAC-DM outperforms SOTA models, particularly on tissue classes, even small and under-represented ones, while achieving substantially faster inference.

## Methodology

**Fig. 1:**
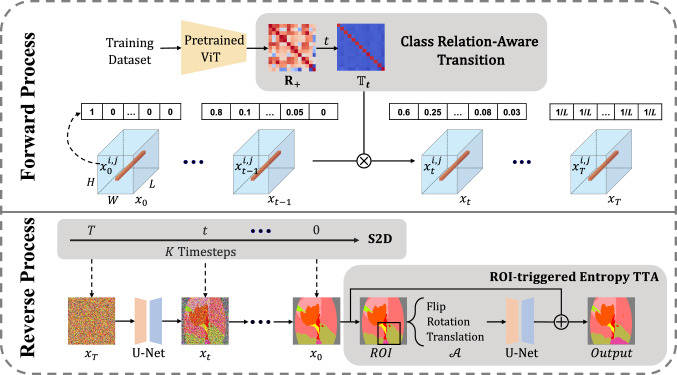
Outline of CRAC-DM. Conditioning image and corresponding extracted features fed into the U-Net in the reverse process are omitted for clarity

Our CRAC-DM consists of a forward process and a reverse process. The outline of our method is shown in Fig. 1.

### Background

Categorical diffusion segmentation models are trained via a forward process that corrupts a clean segmentation map $x_0$ into a sequence of noisy states $x_t$ and a reverse process that recovers $x_0$ over $T$ timesteps. In this categorical setting, each pixel in $x_t$ is modeled as a probability distribution over $L$ classes, i.e., $x_t \in \mathbb {R}^{H \times W \times L}$ [7].

In the forward process, the conditional probability of transitioning from $x_{t-1}$ to $x_t$ is:

1 $$\begin{aligned} q\bigl (x_t \mid x_{t-1}\bigr ) = (1-\beta _t)\,x_{t-1} + \beta _t\,\textbf{U}\,, \end{aligned}$$

where $\beta _t$ is a noise scheduler that increases with step $t$ and $\textbf{U}\in \mathbb {R}^{H \times W \times L}$ with all entries equal to $\tfrac{1}{L}$, representing uniform noise across all *L* classes.

In the reverse process, a neural network (i.e. U-Net) processes the noisy label distribution $x_t$ alongside conditioning features $\textit{f}$ that are extracted from the input image via a pretrained encoder. This produces the probability distribution $p_\theta (x_0 \mid x_t)$ over the original segmentation. Rather than using this predicted distribution directly as the final segmentation result, $p_\theta (x_0 \mid x_t)$ is incorporated into the reverse process to recover $x_{t-1}$, mirroring the step-by-step forward process:

2 $$\begin{aligned} p_\theta \bigl (x_{t-1}\mid x_t\bigr ) \approx \int q\bigl (x_{t-1}\mid x_t, x_0\bigr )\, p_\theta \bigl (x_0\mid x_t\bigr ) \,dx_0, \end{aligned}$$

where $q(x_{t-1}\mid x_t, x_0)$ denotes the categorical posterior:

3 $$\begin{aligned} q\bigl (x_{t-1}\mid x_t, x_0\bigr ) \propto q\bigl (x_t\mid x_{t-1}\bigr )\, q\bigl (x_{t-1}\mid x_0\bigr ). \end{aligned}$$

During training, $x_0$ is the ground truth segmentation map and $q\bigl (x_{t-1}\mid x_t, x_0\bigr )$ is estimated using the forward process in Eq. (1). Since the ground truth $x_0$ is unavailable at inference, the model relies on its learned distribution $p_\theta (x_0\mid x_t)$ as a substitute for $x_0$ at each reverse step. The KL divergence between $p_\theta (x_{t-1}\mid x_t)$ and $q(x_{t-1}\mid x_t, x_0)$ serves as the training loss:

4 $$\begin{aligned} \mathcal {L}_{\textrm{KL}} = \sum _{x_{t-1}} q(x_{t-1}\!\mid x_t,x_0) \log \frac{q(x_{t-1}\!\mid x_t,x_0)}{p_\theta (x_{t-1}\!\mid x_t)}. \end{aligned}$$

### Class relation-aware forward process

In this work, we propose a relation-aware forward process where class labels transition through semantically similar classes, producing more meaningful noisy states $x_t$ that ultimately improve final segmentation accuracy. Specifically, our model leverages class representative features extracted from a pretrained Vision Transformer (ViT) encoder [11] to capture semantic relationships between classes. For each training image *n*, ViT produces a token feature map $\textbf{F}_n \in \mathbb {R}^{H_p \times W_p \times D}$, where each location (*i*, *j*) on the map corresponds to a *D*-dimensional token vector.

Let $Y_n \in \{1,\dots ,L\}^{H \times W}$ denote the groundtruth segmentation mask of image *n* with *L* semantic classes. To align labels with ViT tokens, we resize $Y_n$ to the token-grid resolution using nearest-neighbor interpolation, obtaining $\tilde{Y}_n \in \{1,\dots ,L\}^{H_p \times W_p}$. For each class *c*, we collect all vectors with label *c* into a set:

5 $$\begin{aligned} S_c = \left\{ \textbf{F}_n(i,j)\ \vert |\ \tilde{Y}_n(i,j)=c,\ \forall n,i,j \right\} . \end{aligned}$$

The representative feature vector of class *c*, denoted as $\textbf{f}_c \in \mathbb {R}^{D}$, serves as a global semantic prototype and is computed as the mean of $S_c$:

6 $$\begin{aligned} \textbf{f}_c = \frac{1}{|S_c|}\sum _{\textbf{v}\in S_c}\textbf{v},\quad c\in \{1,\dots ,L\}, \end{aligned}$$

where $\textbf{v}$ denotes a token feature vector (i.e., an element of $S_c$).

We next compute a class similarity matrix $\textbf{R} \in \mathbb {R}^{L \times L}$ using cosine similarity between all class pairs:

7 $$\begin{aligned} \textbf{R}_{c,c'} = \frac{\textbf{f}_{c}\,\cdot \, \textbf{f}_{c'}}{\Vert \textbf{f}_{c}\Vert _2\Vert \textbf{f}_{c'}\Vert _2}. \end{aligned}$$

We perform row-wise normalization on $\textbf{R}$ to obtain $\textbf{R}_{+}$, ensuring that each row sums to one and can serve as a valid probabilistic prior.

In the standard categorical model, the forward process in Eq. (1) is approximated by applying a transition matrix $\mathbb {T}_t = (1-\beta _t)\,\textbf{I} + \beta _t\,\textbf{U}$ to $x_{t-1}$ to obtain $x_t$, where $\textbf{I}$ is the identity matrix and $\textbf{U}$ is the uniform noise matrix (each entry is $\tfrac{1}{L}$). Then, $q\bigl (x_t \mid x_{t-1}\bigr ) = \mathbb {T}_t\,x_{t-1}$. In our work, we incorporate class relation awareness into the forward process by introducing a novel transition matrix:

8 $$\begin{aligned} \mathbb {T}_t = (1-\beta _t)\,\textbf{I} ~+~ \beta _t \Bigl [ (1-\beta _t)\,\textbf{R}_{+} ~+~ \beta _t\,\textbf{U} \Bigr ]. \end{aligned}$$

Notably, class relation awareness is implicitly preserved in the reverse process, as the conditional transition probabilities in Eq. (3) use our proposed transition matrix.

### S2D reverse process with ROI-triggered entropy TTA

#### S2D reverse process

The standard categorical reverse process in Eq. (3) iterates through all $T$ timesteps, which is computationally expensive. Although several accelerated samplers such as DDIM [12] have been proposed, there is no method tailored for discrete diffusion segmentation, especially one which is compatible with customized forward transitions. We therefore propose S2D for efficient inference.

Given the class relation-aware forward transition matrix $\mathbb {T}_t$ in Eq. (8), we first derive the cumulative transition from time $t_a$ to $t_b$ ($t_a < t_b$). This effectively creates a direct probabilistic bridge between non-adjacent steps:

9 $$\begin{aligned} \mathbb {T}^{\,t_a\rightarrow t_b} = \prod _{s=t_a+1}^{t_b}\mathbb {T}_s, \quad \text {so that}\quad q(x_{t_b}\mid x_{t_a}) = \mathbb {T}^{\,t_a\rightarrow t_b}\,x_{t_a}. \end{aligned}$$

To recover the earlier state $x_{t_a}$ from a later noisy state $x_{t_b}$, we apply Bayes’ rule. Extending Eq. (3), the categorical posterior is formulated as the product of the forward likelihood and the prior:

10 $$\begin{aligned} q(x_{t_a}\mid x_{t_b}, x_0) \propto \underbrace{q(x_{t_b}\mid x_{t_a})}_{\text {Likelihood}}\,\underbrace{q(x_{t_a}\mid x_0)}_{\text {Prior}}. \end{aligned}$$

During inference, since the ground truth $x_0$ is latent, we cannot compute Eq. (10) directly. Instead, we marginalize $x_0$ using the network’s predicted distribution $p_\theta (x_0 \mid x_{t_b})$. This computes the expected transition to the previous state:

11 $$\begin{aligned} p_\theta (x_{t_a}\mid x_{t_b}) =\sum \nolimits _{x_0} q(x_{t_a}\mid x_{t_b},x_0)\,p_\theta (x_0\mid x_{t_b}). \end{aligned}$$

This gives a deterministic update $x_{t_b}\!\mapsto \!x_{t_a}$, allowing several timesteps to be skipped without stochastic sampling. Hence, $p_\theta (x_0 \mid x_{t_b})$ in Eq. (11) is a soft class-probability map and no stochastic sampling is applied on it (as in the conventional categorical diffusion models). By propagating these continuous probabilities through the reverse chain and only producing hard labels at the final step, we ensure more stable and spatially coherent segmentation results. We subsample *K* timesteps $\{t_i\}_{i=1}^K$ using a cosine scheduler. This reduces inference complexity from *O*(*T*) to *O*(*K*) while enhancing mask stability by eliminating stochastic sampling noise.

#### ROI-triggered entropy TTA

During inference, S2D outputs a probabilistic segmentation map $x_t \in \mathbb {R}^{H \times W \times L}$, where each pixel $v$ corresponds to a class-probability vector $p(v)$, and each element $p_c(v)$ denotes the predicted probability for class $c$. Uncertainty is measured by the Shannon entropy of the posterior distribution:

12 $$\begin{aligned} \mathcal {H}(v) = -\!\sum _{c=1}^{L} p_c(v)\log p_c(v). \end{aligned}$$

High-entropy (i.e., low segmentation confidence) regions typically correspond to ambiguous tissues or fuzzy boundaries. Previous diffusion-based segmentation models often improve robustness by ensembling multiple stochastic reverse runs, which is computationally expensive. In our S2D, random initial noise introduces negligible variance. Therefore, we design a lightweight, localized, and ROI-triggered entropy TTA.

Pixels with $\mathcal {H}(v)>\tau _h$ (entropy threshold) are marked as uncertain and grouped into connected components. We retain the most uncertain ROI (typically one or a few connected regions per image) and ignore tiny isolated pixels. A small set of augmentations $\mathcal {A}$ (e.g., flips, rotations, or translations) is applied to each ROI together with the original prediction, treated as the identity transformation $a_0$.

For each augmentation $a \in \{a_0\}\cup \mathcal {A}$, we obtain a posterior probability vector $p^{(a)}(v) = [p^{(a)}_1(v), \dots , p^{(a)}_L(v)]$, where $p^{(a)}_c(v)$ denotes the probability of class $c$ at pixel $v$ under augmentation $a$. The corresponding pixel-wise entropy, defined in Eq. (12), is denoted as $\mathcal {H}^{(a)}(v)$. The final prediction for each class is then computed via entropy-weighted averaging:

13 $$\begin{aligned} \tilde{p}_c(v)= \frac{\sum _{a\in \{a_0\}\cup \mathcal {A}} e^{-\mathcal {H}^{(a)}(v)}\,p^{(a)}_c(v)}{\sum _{a\in \{a_0\}\cup \mathcal {A}} e^{-\mathcal {H}^{(a)}(v)}}, \quad v\in \text {ROI}. \end{aligned}$$

This ROI-triggered strategy refines uncertain boundaries and improves prediction consistency, particularly for ambiguous tissue classes, while requiring only a few localized augmentations ($|\mathcal {A}|\!\le \!4$) and ensuring that the original, unaugmented prediction also contributes to the ensembled result.

## Result

### Dataset

We evaluate our model on two public surgical scene datasets, namely CholecSeg8k [9] and EndoVis18 [10]. CholecSeg8k consists of 8080 laparoscopic cholecystectomy frames distributed across 101 video clips. We split these into 81 clips for training (6480 images) and 20 clips for testing (1600 images). EndoVis18 contains 3232 images captured during 19 kidney and small intestine procedures on porcine models. These sequences were split into 15 training sets and 4 test sets. Each sequence comes from a single porcine procedure, and the test set contains procedures different from the training set. We follow the official train/test split provided by the challenge, without applying any additional stratification, and use all provided test sets for evaluation. For computational efficiency, all images are resized to $256\times 256$.

### Implementation detail

All experiments are conducted on an NVIDIA RTX A6000 GPU. We benchmark our approach against SOTA models, including U-Net-based (nnU-Net [13]), transformer–U-Net hybrid (TransUNet [14]), transformer-based (SegFormer [15], Mask2Former [16]), and two diffusion-based methods (MedSegDiffv2 [4], CCDM [7]), with CCDM serving as the primary baseline on which our model is developed. MedSegDiffv2, CCDM, and nnU-Net are trained using their default configurations. For all other comparison models, we use a batch size of 64, train for 80,000 iterations (i.e. 790 epochs for CholecSeg8K and 2290 for EndoVis18, respectively) and keep other default hyperparameters.

Our proposed CRAC-DM and all ablation variants are trained using the Adam optimizer [17] with an initial learning rate of $1\times 10^{-4}$, a batch size of 16, and for 80,000 iterations (i.e. 198 epochs for CholecSeg8K and 572 for EndoVis18, respectively). We employ a cosine noise scheduler over 250 full training timesteps. The proposed S2D sampler with 10 timesteps is applied only at test time to accelerate inference. To derive class relationships, we use the pretrained DINOv2-base transformer [18] as the encoder, while the conditional feature extractor (which provides raw image features) is based on the pretrained DINO-base [19], identical to that used in CCDM. This setup ensures a fair comparison by guaranteeing that any performance gains relative to CCDM are not due to differences in the conditioning feature extractor.

We set the number of reverse denoising steps $K = 10$, and use augmentations $\mathcal {A}=\{$horizontal and vertical flips, rotations by $90^\circ$ and $270^\circ$, and random small translations of up to $\pm 10$ pixels$\}$. For the ROI, we implement the selection process by applying 8-connectivity Connected Component Labeling to the uncertainty mask ($H(v) > 0.3$), where regions smaller than 100 pixels are filtered out and only the single largest component is retained. For performance evaluation, we report the Intersection over Union (IoU) for each class and use the mean IoU (mIoU) as the primary metric. Due to space constraints, we report only classes with sufficient representation, while near-empty classes and the black background are omitted.

**Table 1:** Comparison of segmentation models on CholecSeg8K and EndoVis18

CholecSeg8K												
Model	Per-Class IoU (%) $\uparrow$											mIoU (%) $\uparrow$
	Abd.	Liver	GI	Fat	Grasp.	Con. Tiss.	Blood	Cyst. D.	L-hook	Gallbl.	Liver Lig.	
nnU-Net	80.13	92.41	59.39	90.46	70.45	76.70	*55.19*	**67.73**	84.56	80.13	**96.98**	77.65
SegFormer	87.43	86.91	64.71	86.88	69.48	67.91	45.06	32.67	71.37	76.16	96.22	71.35
Mask2Former	92.83	91.70	69.09	88.25	81.12	*80.39*	53.59	44.74	87.53	83.83	*96.86*	79.09
TransUnet	81.03	89.85	68.38	87.85	67.18	79.07	46.27	*65.40*	85.59	80.94	96.04	78.49
MedSegDiffv2	81.31	89.19	62.77	89.05	68.93	77.68	42.73	52.73	83.68	81.44	96.84	75.12
CCDM	*93.61*	*92.63*	*78.01*	*90.88*	*83.34*	79.40	55.01	57.82	*88.31*	*84.29*	96.73	*81.81*
CRAC-DM	**93.92**	**93.93**	**82.47**	**92.89**	**87.58**	**82.04**	**62.49**	64.96	**90.62**	**85.97**	95.86	**84.79**

EndoVis18												
Model	Per-Class IoU (%) $\uparrow$											mIoU (%) $\uparrow$
	Bkg Tiss.	Shaft	Clasp.	Wrist	Kid. Paren.	Cov. Kid.	Thread	Clamps	Intest.	US Probe		
nnU-Net	*78.56*	74.45	42.37	40.92	54.12	17.89	4.87	**28.97**	19.36	8.68	37.02	
SegFormer	63.11	77.63	36.96	41.88	47.33	20.26	0.32	0.41	29.43	8.60	32.59	
Mask2Former	73.59	**86.27**	**51.85**	**61.24**	*71.45*	30.27	7.40	11.14	42.07	24.71	46.00	
TransUnet	**81.58**	85.47	50.80	*51.51*	59.81	*33.22*	7.86	16.59	*45.94*	**32.75**	46.55	
MedSegDiffv2	68.65	80.73	40.37	47.58	48.55	11.00	16.51	20.90	15.26	1.44	35.10	
CCDM	74.76	*86.15*	47.45	*58.06*	69.86	30.75	**20.40**	*24.63*	39.28	21.50	*47.28*	
CRAC-DM	75.31	85.32	46.47	56.96	**73.73**	**36.10**	*17.71*	24.31	**45.96**	*25.57*	**48.74**	

Best results are highlighted in bold, and second-best results are italics

### Performance evaluation

In Table 1, our proposed CRAC-DM consistently outperforms all SOTA baselines on the CholecSeg8k dataset, where most categories (except the grasper and L-hook) correspond to tissues. Specifically, CRAC-DM achieves a +2.98% mIoU improvement over the second-best CCDM and +5.70% mIoU over Mask2Former. Notably, both CRAC-DM and CCDM are built upon the same medium-sized U-Net backbone. The fact that CCDM ranks second demonstrates the strong representational power of discrete diffusion models for surgical image segmentation. CRAC-DM further achieves substantial gains beyond the backbone’s capacity.

Per-class analysis shows that CRAC-DM has the highest IoU in 9 out of 11 categories. Especially for the small and highly variable blood class (present in 8.56% of images and covering 4.93% of pixels on average), our model improves IoU by +7.30% compared with the second-best. For the clinically important yet highly under-represented cystic duct (present in only 3.07% of images and occupying 1.20% of the area on average), CRAC-DM achieves a +7.16% IoU gain over CCDM.

For the EndoVis18 dataset (Table 1), which mainly consists of surgical instrument classes, CRAC-DM also achieves SOTA performance. Specifically, it exceeds CCDM by +1.46% mIoU and TransUNet by +2.19% mIoU overall. Notably, among the tissue classes, CRAC-DM achieves the best IoU in kidney parenchyma, covered kidney, and intestine, surpassing the respective second-best methods by +2.28%, +2.88%, and +0.02%, respectively. When aggregating only tissue-related categories, CRAC-DM outperforms CCDM and TransUNet by +4.12% and +2.64% mIoU, respectively. These findings suggest that although CRAC-DM achieves relatively smaller improvements on the instrument-heavy dataset, its class-aware transition and targeted refinement still provide consistent advantages for segmenting challenging tissue, making the model well suited for surgical scenes.

**Fig. 2:**
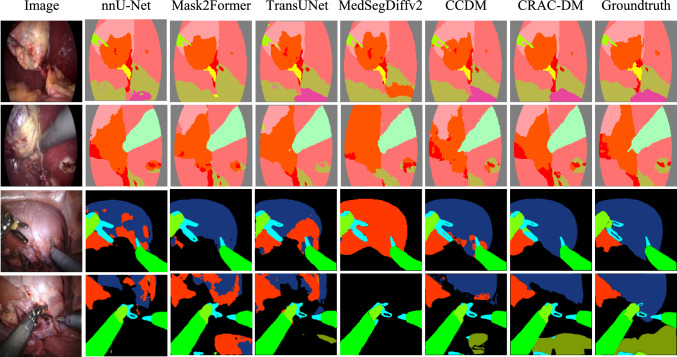
Performance evaluation of the compared models on the CholecSeg8k (top two rows) and EndoVis18 datasets (bottom two rows)

Qualitative results in Fig. 2 further highlight this advantage, showing that CRAC-DM produces more accurate delineations, particularly for ambiguous or under-represented tissue classes that remain challenging for existing methods. In the first row, CRAC-DM achieves the most accurate segmentation of the blood class (red) and the gastrointestinal tract (purple). In the second row, although CRAC-DM performs slightly worse than CCDM on the tip of the shiny green L-hook, it delineates more accurately the red blood regions, which are notoriously difficult to segment due to their highly variable appearance and lack of a consistent shape. The bottom two rows are from the EndoVis18 dataset. While all models yield comparable segmentation results for surgical tools, CRAC-DM provides the most consistent and precise segmentation of the tissue classes namely, kidney parenchyma (red), covered kidney (dark blue), and intestine (green).

### Ablation study

To thoroughly validate CRAC-DM, we conduct a comprehensive ablation study. We first present the cumulative effectiveness of our three core contributions, namely Class Relation-aware Forward Process, S2D Reverse Process, and ROI-triggered TTA. Subsequently, we provide a detailed analysis of each component’s impact.

#### Overall effectiveness

**Table 2:** Ablation study on CholecSeg8k

Relation-aware	Reverse steps (*K*)	TTA	mIoU (%) $\uparrow$	mDice (%) $\uparrow$	Boundary mIoU (%) $\uparrow$
✗	250	✗	81.81	89.36	60.90
✓	250	✗	83.58	90.57	62.60
✓	10	✗	84.22	90.95	63.82
✓	10	✓	**84.79**	**91.37**	**64.70**

Best results are in bold

Table 2 summarizes the step-by-step performance gains. The baseline, which uses a uniform transition matrix and $K=250$, achieves an mIoU of 81.81%.

**Step 1 (Relation awareness):** Replacing the uniform transition with class relation-aware transition yields the most significant individual gain of +1.77% mIoU.

**Step 2 (Jump Sampling):** Switching to the deterministic S2D with steps ($K=10$) further improves mIoU to 84.22% (+0.64%). The jump sampling strategy not only accelerates inference but also enhances prediction accuracy.

**Step 3 (ROI-TTA):** Finally, integrating the ROI-triggered entropy TTA gives the peak performance of 84.79% mIoU and 91.37% mDice.

Notably, the addition of each component consistently improves boundary mIoU (from 60.90 to 64.70%). This indicates that the improvements are critical for correcting fine-grained errors at class boundaries.

#### Class relation-awareness ablation

**Fig. 3:**
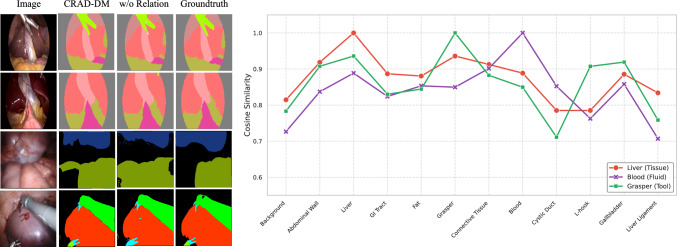
Class relation analysis. Left: Segmentation results with and without class relations. Right: Representative class relation curves on CholecSeg8k

**Table 3:** Efficiency analysis on CholecSeg8k

Method	Reverse steps (*K*)	mIoU (%) $\uparrow$	TFLOPs $\downarrow$	FPS $\uparrow$
CCDM	250	81.81	8.41	4
CRAC-DM	250	84.10	8.41	2
CRAC-DM	50	84.31	1.68	8
**CRAC-DM**	**10**	**84.79**	**0.34**	**30**
CRAC-DM	5	84.54	0.17	43
CRAC-DM	2	83.86	0.07	64

FLOPs are estimated as $K \times$ single U-Net forward cost without TTA. FPS is measured using the full inference pipeline with TTA enabled. Best trade-off highlighted in bold

To analyze the class relationships learned by our model, Fig. 3 (Right) presents the relation values in *R* for the liver, blood and grasper classes. Notably, the liver exhibits strong connections not only with itself but also with the abdominal wall, connective tissue, and grasper. This indicates that the learned similarity captures more than just low-level visual features. It also contains high-level semantic context, such as spatial information and class interactions. Similarly, the grasper shows high affinity with the L-hook, liver, and gallbladder. This reflects interactions in real surgical scenarios where graspers frequently manipulate the liver. Class blood demonstrates strong similarity with vascularized tissues but low affinity with the liver ligament. These relations constrain the forward process to induce more realistic noise patterns during training.

Visual comparisons in Fig. 3 (Left) further verify the importance of class relation-awareness. In the first row (CholecSeg8k), the model without relation-awareness confuses the semantically similar abdominal wall (light gray) with liver (dark pink). CRAC-DM resolves this ambiguity, producing a nearly perfect segmentation. In the second row, the model without relation-awareness predicts fragmented grasper segmentations (green spots) within the liver (dark pink). CRAC-DM successfully eliminates these isolated noise patterns. On the bottom two rows (EndoVis18), CRAC-DM continues to demonstrate superior performance in distinguishing similar tissues. It also effectively prevents the appearance of isolated segmented areas, proving the robustness of the relation-aware prior.

#### Jump sampling ablation

In Table 3, we analyse the trade-off between the number of reverse steps (*K*) and performance.

**Accuracy:** Reducing steps from 250 to 10 improves mIoU (84.10% $\rightarrow$ 84.79%). We attribute this to the deterministic nature of our jump sampling (S2D), which avoids the error accumulation inherent in long sampling chains.

**Real-time Capability:** Crucially, this reduction unlocks real-time performance. While the CRAC-DM with $K=250$ operates at 2 FPS (8.41 TFLOPs), our method at $K=10$ achieves 30 FPS with 0.34 TFLOPs. This 15$\times$ speedup verifies jump sampling as a vital architectural contribution, allowing high-quality diffusion segmentation to meet clinical latency requirements. It is also worth noting that for extremely few steps ($K=5$ or $K=2$), the model maintains significant accuracy, offering a flexible trade-off for applications prioritizing high FPS.

**Table 4:** ROI-triggered entropy TTA ablation

ROI strategy	Fusion	$\tau _h$	mIoU (%) $\uparrow$
–	–	–	84.22
Full image	Simple avg	–	84.40
ROI	Simple avg	0.3	84.49
ROI	Entropy-weighted	0.1	84.55
**ROI**	**Entropy-weighted**	**0.3**	**84.79**
ROI	Entropy-weighted	0.5	84.60

Our choice is highlighted in bold

#### ROI-triggered TTA ablation

According to Table 4, our ROI-triggered TTA with entropy-weighted fusion ($\tau _h=0.3$) achieves a peak 84.79% mIoU. By applying augmentation specifically to high-entropy regions, the model resolves ambiguous segmentations, especially at class boundaries while preserving uniform predictions in homogeneous areas. The entropy-weighted fusion serves as a reliability-based aggregation, outperforming simple averaging by 0.30%. While performance is robust across $\tau _h \in [0.1, 0.5]$, the 0.3 threshold provides the best trade-off between local refinement and global consistency.

## Conclusion

In this work, we introduced the CRAC-DM for surgical scene segmentation. Our model enhances discrete diffusion by incorporating inter-class similarity into the forward diffusion process and accelerating inference through the novel deterministic S2D. A lightweight ROI-triggered entropy TTA further refines boundaries with minimal cost. Experiments on CholecSeg8k and EndoVis18 demonstrate consistent gains over SOTA baselines, particularly on ambiguous tissue classes. Overall, CRAC-DM provides an accurate, robust, and efficient framework for handling the complexity of surgical scene segmentation.

Promising future directions include integrating discrete score-based formulations (e.g., SEDD [20]) on language modeling tasks to enhance discrete denoising, adopting uncertainty-aware probabilistic modeling (e.g., Dirichlet–multinomial) to improve robustness for rare classes, and evaluating performance in surgical robotic deployment settings. The applicability of CRAC-DM to other medical modalities (e.g., MRI and CT) and broader vision datasets will also be explored.
